# A systematic review of clinical psychological guidance for perinatal mental health

**DOI:** 10.1186/s12888-023-05173-1

**Published:** 2023-10-30

**Authors:** Jayne O’Brien, Lynsey Gregg, Anja Wittkowski

**Affiliations:** 1 The University of Manchester, School of Health Sciences, Manchester, M13 9PL UK; 2 Greater Manchester Mental Health NHS Foundation Trust, Manchester, M25 3BL UK; 3 The University of Manchester, Manchester Health Alliance Science Centre, Manchester, M13 9PL UK; 4 Division of Psychology and Mental Health, School of Health Sciences, Faculty of Biology, Medicine and Health, The University of Manchester, 2nd Floor Zochonis Building, Brunswick Street, Manchester, M13 9PL UK; 5 Manchester Academic Health Science Centre, Manchester, M13 9NQ UK

**Keywords:** Mothers, Women, Parents, Mental health, Guidelines, Psychosocial, Therapy, Intervention, Assessment

## Abstract

**Background:**

Guidelines on psychological and/or psychosocial assessment and intervention in the perinatal period can provide beneficial practice guidance for healthcare professions to reduce maternal distress and potential mortality. As little is known about the similarities in recommendations across guidelines, which could impact the quality of therapeutic intervention women receive, this systematic review was conducted to draw out the consistent guidance for perinatal psychological and/or psychosocial therapeutic input.

**Method:**

Eight literature and two guideline databases were searched alongside guideline development institutions, and organisations of maternity or perinatal mental health care. All relevant guidance was searched for and extracted before guideline quality was assessed using the AGREE-II instrument. Included guidelines had a primary or secondary focus on psychological assessment and therapeutic intervention for perinatal mental health difficulties. Using a narrative synthesis approach, recommendation consistencies and inconsistencies were outlined.

**Results:**

From the 92 records screened, seven guidelines met the inclusion criteria. Only two guidelines were rated high (> 80%) across all assessed domains, with the other guidelines scoring between poor and excellent across domains. Highest rated domains across all seven guidelines were *clarity of presentation* (75%) and *scope and purpose* (70%). Recommendations for structured psychological assessment and intervention were most commonly reported in the guidelines; however, the level of detail and depth of information varied across guidelines. Whilst assessment and intervention recommendations for mother-infant dyad and partners were considered, research into working therapeutically with these client groups in perinatal mental health services is only just emerging. Hence, guideline recommendations for working with the mother-infant dyad and partners were based on consensus of expert opinion.

**Conclusion:**

Perinatal mental health guidelines were consistent in scope but showed considerable variability in quality and depth of recommendations, which could have implications for standards of clinical practice. However, there is still a need to improve the evidence underpinning recommendations in perinatal mental health guidelines to advance the implementation of psychological and/or psychosocial interventions. High quality interventions in the perinatal period could improve outcomes for women and their families.

**Supplementary Information:**

The online version contains supplementary material available at 10.1186/s12888-023-05173-1.

## Introduction

The perinatal period, defined here and in the NHS Long Term Plan [1] as the timeframe between pregnancy to two years after birth, can be a time of enormous change. This transition to motherhood can impact the woman’s mental health and in unexpected ways. For example, the arrival of a new baby can trigger relationship strains [2] or unresolved psychological trauma [3, 4]; it can impact a mother’s self-efficacy [5] and lead to changes in identity by distorting then reconstructing her sense of who she is [6].

Perinatal psychological distress (PPD) involves an adverse and prolonged emotional state, which can be reflected in a person’s behaviour, possibly causing them harm or adversely affecting their relationships [7]. PPD affects 21.2% of women prenatally and 26.7% postnatally [8]. A range of mental health difficulties, such as anxiety and depression, are typically seen with PPD in response to specific psychosocial stressors [9, 10].

Internationally, around 10–13% of women experience perinatal mental health difficulties (PMHDs) [11]. Anxiety and depression are the most common PMHDs, affecting 13% of women in high income countries [12, 13], 15–20% of women in low- and middle-income countries [14] and 42% of migrant women in the UK [15]. Postnatal anxiety tends to be more prevalent than postnatal depression in high-income countries [14], whereas higher rates of postnatal depression have been observed in low- to middle-income countries [16]. However, anxiety and depression often co-exist [17, 18] or are comorbid with other mental health conditions, such as post-traumatic stress disorder [19, 20].

The current evidence base for psychological therapies in the perinatal period is limited to cognitive behavioural therapy (CBT) and interpersonal psychotherapy (IPT) [21–23], which are the recommended psychological interventions in a perinatal competency framework for UK services [24]. Controlled trials are often restricted to particular PMHDs, such as anxiety or depression, so the evidence-base tends to be diagnosis-specific. Other therapies, such as acceptance and commitment therapy (ACT, see Hayes et al. [25]; for perinatal adaptation see Bonacquisti et al. [26]) and compassion-focused therapy (CFT, see Gilbert [27]; for perinatal adaptation see Cree [28]), are offered across perinatal community mental health teams in the UK [29], but these interventions have not yet received much research attention. Further higher quality studies, including systematic reviews and controlled trials on other types of psychological interventions (e.g., third wave approaches), are required [21].

Globally, PMHDs are considered a major public health challenge [30], because they significantly impact the mother, baby and family, and can potentially be fatal [31–35]. In Ireland and the UK, suicide is the leading cause of maternal death in the perinatal period [34, 36] and, although rare, another potential consequence of PMHDs is neonaticide or infanticide [37, 38].

PMHDs are known to affect the bond between mother and baby, which can influence the psychological wellbeing and development of the infant, as seen in delayed and poorer cognitive, social and emotional development [39–42]. There is growing evidence to suggest interventions focusing on mother-infant interactions benefit the dyadic relationship [43] as well as strengthening attunement during a crucial stage of infant brain development [44]. Furthermore, partners are also more likely to experience mental health difficulties in the perinatal period [45]; they frequently report feeling overwhelmed, lonely and frustrated in relation to maternal PMHDs [46].

In the UK, estimated benefits of perinatal service provision during 2015 and 2020 were approximately £21million across both years [47]. According to Bauer et al. [47], if perinatal mental health needs could be met through an integrated model of primary care services in the UK, then estimated benefits would be £70 million.

It has now been widely recognised that high quality perinatal mental health care can improve the immediacy and accessibility of support for mothers [48–52]. Specialist mental health services adapted for the perinatal period are highly valued by women and they aree usually preferred to generic mental health services [53]. Practice guidelines can set a benchmark by defining the optimum quality of clinical care for perinatal mental health. For the purpose of this review, clinical practice guidelines were defined as “statements that include recommendations, intended to optimise patient care, that are informed by a systematic review of evidence and an assessment of the benefits and harms of alternative care options” [54, p.15]. Adherence to these best practice standards is a measurable objective for quality improvement efforts [55, 56] and can result in earlier gains in psychological intervention plus shorter treatment times [57–59].

To date, reviews of clinical practice guidelines have focused on perinatal health care in its broadest sense. Haran et al. [60] and Yang et al. [61] reviewed postpartum health and mental health care guidelines for routine clinical practice. Both reviews concentrated on postpartum health care only, with mental health being part of that wider focus. Haran et al. [60] reviewed only the Scottish Intercollegiate Guidelines Network (SIGN [62*]) guideline, whereas Yang et al. [61] included three mental health guidelines (SIGN [62*]; Registered Nurses Association of Ontario-RNAO [63*]; National Institute of Clinical Excellence-NICE [64*]). However, both did not include relevant perinatal guidelines (e.g., Australia’s Centre of Perinatal Excellence -COPE [65*]), published within their search timeframe (2002–2021). Furthermore, neither review focused on psychological and/or psychosocial assessment beyond routine screening, nor did they include specifics about facilitation of psychological interventions within specialist perinatal mental health settings.

Guidelines can bridge the gap between policy, best practice and local contexts, helping to ensure consistent care across specialist mental health settings [66]. A comprehensive review can assist in the potential refinement of policies – local, national or international – and to advance the implementation of perinatal psychological and psychosocial interventions. This systematic review aimed to 1) collate and summarise available clinical guidance on perinatal psychological mental health care, 2) explore the consistency of guideline recommendations made for the psychological and/or psychosocial assessment and treatment of women, the mother-infant dyad and, if possible, partners during the perinatal period, and 3) summarise the key recommendations for the psychological assessment and treatment of women and, if possible, the mother-infant dyad and partners.

## Method

The review protocol was registered with the International Prospective Register of Systematic Reviews (PROSPERO) in September 2020 (registration number CRD202183). Johnston et al.’s [67] methodological guidance on conducting systematic reviews of clinical practice guidelines was followed because these types of reviews require tailored approaches to and greater subjectivity in design and execution compared with other systematic reviews. The manuscript was prepared in accordance with the PRISMA checklist for systematic reviews and meta-analysis [68].

### Search strategy

The database searches were performed by the first author in August 2022. Due to the advances in perinatal mental health research and clinical practice over the last ten years, guidelines published between 2012 and 2022 were sought. We searched eight international bibliographical databases, namely PubMed, EMBASE, MIDIRS, CINAHL, PsycINFO, MEDLINE, Social Policy and Practice, and Web of Science, alongside two specific guideline databases including the *Guidelines International Network* and *Guideline Central*. In addition, websites of relevant healthcare professional bodies in English speaking countries were also searched (e.g., the British Psychological Society, the Royal College of Psychiatrists, the American Psychological Association, the American Psychiatric Association, Health Service Executive (HSE) in Ireland, Health and Social Care (HSC) public health in Northern Ireland, etc.).

The search included combinations of the terms ‘maternal’, ‘perinatal’, ‘postpartum’, ‘peripartum’, ‘antenatal’, ‘postnatal’ and ‘mental*’. Search results were filtered by publication type and/or subject type (e.g., ‘clinical guidelines’, ‘practice guidelines’ or ‘guideline’) depending on the filters available across databases (see search strategy in Table 1). Furthermore, the *Guideline Central* based in the United States and *Guidelines International Network* were searched but no limits were needed for practice or clinical guidelines due to the nature of the database. Search terms were entered into the databases individually and combined with ‘mental*’ to ensure greater breadth of our results.

**Table 1:** Search terms per database

Search block	Medline / Embase / PSYCHINFO / Social Policy and Practice / MIDRS via OVID	CINAHL PLUS via EBSCO	PubMed	Web of Science	Guidelines International Network	Guideline Central
**Population**	**Maternal.ti** **OR** **Perinatal.ti** **OR** **Postpartum.ti** **OR** **Peripartum.ti** **OR** **Antenatal.ti** **OR** **Postnatal.ti**	**Maternal** **OR** **Perinatal** **OR** **Postpartum** **OR** **Peripartum** **OR** **Antenatal** **OR** **Postnatal**	**Maternal** **OR** **Perinatal** **OR** **Postpartum** **OR** **Peripartum** **OR** **Antenatal** **OR** **Postnatal**	**Maternal.ti** **OR** **Perinatal.ti** **OR** **Postpartum.ti** **OR** **Peripartum.ti** **OR** **Antenatal.ti** **OR** **Postnatal.ti**	1. Perinatal mental* 2. Antenatal mental* 3. Postnatal mental* 4. Peripartum mental* 5. Postpartum mental* 6. Maternal mental* **7. perinatal** **8. peripartum** **9. maternal** **10. postnatal** **11. antenatal**	1. Perinatal mental* 2. antenatal mental* 3. postnatal mental* 4. peripartum mental* 5. postpartum mental* 6. maternal mental* **7. perinatal** **8. peripartum** **9. maternal** **10. postnatal** **11. antenatal**
***AND***						
	Mental*.ti **Guideline.ti**	**-**	Mental*	**Mental health.ti** **AND Guidelines.ti**	**-**	**-**
***LIMITERS / FILTERS APPLIED***						
**Limits applied**	**-**	**Publication type:** **Practice guidelines** **Subject major heading:** **1. Practice guidelines** **2. Mental disorders** **3. Behavioural and mental disorders**	**Article type:** **1. Guideline** **2. Practice Guideline**	**-**	**-**	**-**

Key: (-) function not available or not used

### Identification and selection of guidelines

The PICAR framework was used to guide the inclusion and exclusion criteria (Table 2). Once all searches were completed, the first author and a second reviewer (AR) screened for eligibility based on title and abstract using pre-determined criteria. Some guidelines did not provide an abstract so potentially relevant records were assessed in a second stage of screening along with all identified eligible records, for which full texts were obtained, and then screened for inclusion.

**Table 2:** Overview of the PICAR framework and its criteria for inclusion or exclusion of records

PICAR Framework	Eligibility criteria
**P**opulation, clinical indication(s), and condition(s)	**Study population** • Women experiencing perinatal mental health difficulties (PMHDs) • Outpatients/inpatients **Clinical indication** • Psychological/psycho-social assessment and/or intervention for women / mother-baby dyad / partners affected by PMHDs **Clinical Condition** • Perinatal mental health difficulties (defined here as during pregnancy and two years after birth) • For the purposes of this review, all guidelines or guides that focus on PMHDs – regardless of definition – were used
**I**nterventions	• Any psychological/psychosocial assessment/intervention for PMHDs
**C**omparator(s), Comparison(s), and (key) content	• Any comparator/comparison is of interest • If guidelines are broader in scope, content specific to PMHDs is only of interest
**A**ttributes of the eligible CPGs	**Language** • Available in English – due to resource limitations **Year of publication** • 2012 onwards **Developing/publishing organisation** • Only guidelines or recommendations issued or endorsed by national and international scientific societies, professional colleges, charitable organisations, healthcare and government organisations were included **Version** • Latest version only **Development process** • Must be explicitly evidence-based **Quality of evidence** • The eligibility of guidelines or guides were not based on a specific minimum quality cut-off score based on the AGREE-II criteria • We are interested in all guidance regardless of quality indicated by the AGREE-II tool **Scope** • Must have a primary/secondary focus on psychological assessment and therapeutic intervention for PMHDs • Covers any aspect of psychological/psychosocial assessment and intervention • Must be clearly defined as clinical practice guidelines, guidance or guide • Must be published
**R**ecommendations	• Must have substantial information on recommendations concerning the psychological/psychosocial assessment and therapeutic intervention for PMHDs in primary and/or specialist care • Supporting evidence for recommendations must be clearly documented and referenced

### Data extraction

The recommendations to be extracted had to focus on psychological or psychosocial assessment and/or intervention for women, the mother-baby dyad and partners affected by PMHDs. Specific recommendation categories (see Supplementary Table 1) were scored as follows: (-) no recommendations provided in the guideline, (✓) some listed, and (✓✓) comprehensive list given. Pertinent findings were drawn out of guideline content and tabulated. The tick scores were then assigned numerical values (e.g., (-) = 0, (✓) = 1, (✓✓) = 2) in Microsoft Excel [69]. A total score was calculated by adding the values horizontally across each recommendation category, then vertically for the target population within the various stages of assessment and intervention. The percentage of coverage for each recommendation category and every target population/stage of intervention were determined by dividing the respective total score by the maximum possible score and multiplying this value by 100. Finally, the mean percentages for the respective target populations (i.e., women, mother-infant dyad, partners) were added together across the four stages of intervention (i.e., antenatal and postnatal assessment and intervention), then divided by four using Microsoft Excel [69]. These findings are presented in the results section.

### Quality assessment

The 23-item AGREE-II instrument [70] was used to appraise methodological quality, because it a) provides a framework to assess the quality of guidelines, b) offers a methodological strategy for the development of guidelines and c) informs what and how information should be reported. AGREE-II uses a 7-point Likert scale of agreement from 1 (strongly disagree/information not present) to 7 (strongly agree). Guidelines were assessed across six domains: scope and purpose, stakeholder involvement, rigour of development, clarity of presentation, applicability and editorial independence. An overall guideline assessment score was assigned by reviewers who also determined whether use of the guideline was recommended (options were ‘yes’, ‘yes with modifications’ or ‘no’).

Each guideline was independently rated by two reviewers (JOB & ZK). To measure interrater agreement across ordinal AGREE-II rating categories, a weighted kappa was calculated using SPSS V.28.0 [71] to assign less weight to agreement as categories were further apart [72]. The weighted kappa values within each AGREE-II domain ranged from moderate κ_w_ = 0.47 (95% CI 0.11 to 0.82) to almost perfect agreement κ_w_ = 0.924 (95% CI 0.861 to 0.987). Overall inter-rater agreement across all AGREE-II item scores was good with an intraclass correlation of 0.87 (95% CI 0.77 to 0.94). Domain scores were calculated by summing up all the scores of the individual items in a domain and by scaling the total as a percentage of the maximum possible score for that domain [70]. To make the scores more accessible to readers and enable comparison, the AGREE II outcomes are reported categorically using the five-point Likert scale described by Yang et al. [61]: excellent (> 80%), good (> 60–80%), average (> 40–60%), fair (> 20–40%) and poor (≤ 20%). All items on the AGREE-II tool were equally weighted but might not have been of equal importance in terms of the richness of guideline content and recommendations. For example, some guidelines might have scored highly on the AGREE-II tool, but the quality and depth of recommendations in the guideline were poor.

### Synthesis

A narrative synthesis of the findings was conducted to describe the scope, content and consistency of the recommendations across guidelines comparable to the approach taken by Hennessy et al. [73]. Initially, guidelines were summarised to gain an overall knowledge of guideline content (see “Overview of each guideline” section), then the text was coded using NVivo version 12 [74] to identify domains of psychological or psychosocial assessment and intervention. The inclusion of specific themes and domains were discussed with the review team for completeness and accuracy. Within each theme, recommendations were further coded into separate categories (e.g., structured psychological approaches, therapeutic relationship in perinatal context, care-planning). Direct comparisons of key recommendations were then tabulated (see Supplementary Tables 2 and 3) to explore their consistency by identifying similarities and discrepancies in content. Finally, common key recommendations across guidelines were summarised.

## Results

**Fig. 1:**
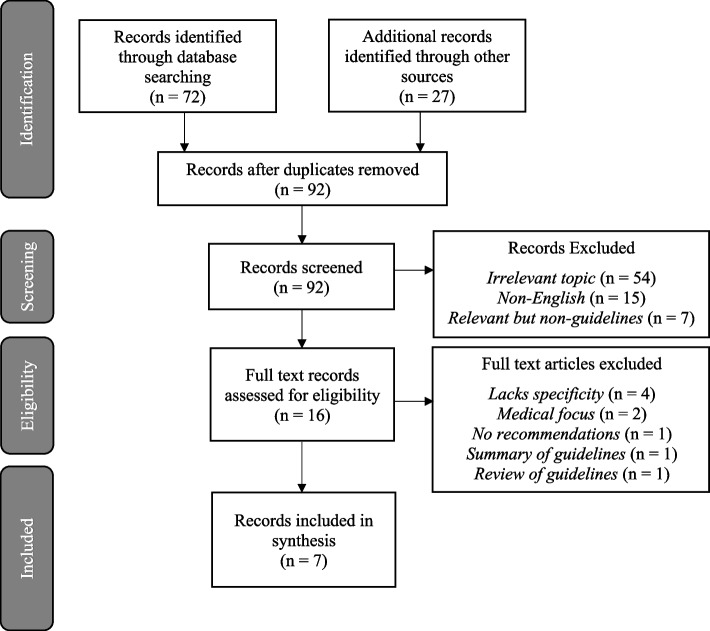
PRISMA diagram outlining identification of guidelines (records selected for synthesis are all guidelines)

A total of 99 records were identified by the database and other source searches, with seven duplicates. After screening and review, seven guidelines were included in the review (Fig. 1). Five of the included guidelines covered perinatal mental health care, one focused on perinatal mental health within a family context and one covered new-born care more broadly.

The characteristics and development processes of each guideline are provided in Table 3. Guideline development groups were from Australia (*n* = 1), England, (*n* = 2), Scotland (*n* = 1), and Canada (*n* = 3). As guidelines published in the United States only focused on perinatal health care with no specific guidance on perinatal mental health care, they were not included in this review.

**Table 3:** Characteristics and scope of the seven included guidelines in chronological order

							**Antenatal Mental Health**			**Postnatal Mental Health**		
No	**Guideline name**	**Guidelines organisation/society/** **authors, year, country**	**Guideline developers**	**Target users**	**Guideline review process**	**Search strategy for evidence**	**Women**	**Mother** **-baby dyad**	**Partner**	**Women**	**Mother-** **baby dyad**	**Partner**
1	(SIGN 127) Management of perinatal mood disorders	Scottish Intercollegiate Guidelines Network (SIGN) (2012), Scotland [62*]	Multi-disciplinary and service-user (or lay) representatives	Healthcare professionals, social services and community agencies, such as voluntary organisations and social services	Independent peer review/ editorial group review	Systematic literature review	✵	**-**	**-**	✵	✵	**-**
2	Mental health disorders in the Perinatal Period	British Columbia (BC) Reproductive Mental Health Program ans Perinatal Services BC (2014), Canada [75*]	Multi-disciplinary	Healthcare professionals	External / internal individual reviewers	Systematic review	✵	✵	✵	✵	✵	✵
3	Mental health conditions in the perinatal period	Centre of Perinatal Excellence (COPE) (2017), Australia [65*]	Multi-disciplinary	Healthcare professionals	External independent methodologists, independent peer review	Systematic literature review	✵	✵	✵	✵	✵	✵
4	Assessment and Interventions for Perinatal Depression – second edition	Registered Nurses’ Association of Ontario (RNAO) (2018), Canada [63*]	Nurses and their interprofessional group	Nurses and other healthcare professionals. Educators, policy makers, persons and their families affected by perinatal mental health difficulties	Public and organisational stakeholder reviewers	Systematic literature review	✵	✵	**-**	✵	✵	**-**
5	Family-centred maternity and new born care – national guidelines Canada	Public Health Agency of Canada (2020), Canada [76*]	Multi-disciplinary	Health care providers other people involved in maternal and newborn health and those who plan, manage and decide on maternal and newborn health programs and services	Internal individual reviewer	Not explicitly stated—? systematic review	✵	✵	✵	✵	✵	✵
6	Antenatal and postnatal mental health: clinical management and service guidance	National Institute for Clinical Excellence (NICE) (2020), England [64*]	Multi-disciplinary and service-user representatives	Health professionals, commissioners, social services, voluntary and private sectors, and women and their families during the perinatal period	Public consultation with registered stakeholders	Systematic literature review	✵	✵	✵	✵	✵	✵
7	Involving and supporting partners and other family members in specialist perinatal mental health services: Good practice guide	NHS England (2021), England [77*]	Multi-disciplinary	Specialist perinatal mental health services and commissioners	Expert reference group review	Systematic literature review	✵	✵	✵	✵	✵	✵

Most guideline developers conducted a systematic review of the literature; however, some (e.g., Reproductive Mental Health Program and Perinatal Services BC [75*]; Public Health Agency of Canada [76*]; NHS England [77*]) did not publish a technical report detailing specifics about their searches of the evidence base. Three guideline developers [78–80] graded both the level of study evidence included for review and the strength of recommendations made, whereas two guidelines (e.g., NICE [64*]; Reproductive Mental Health Program and Perinatal Services British Columbia (BC) [75*]) simply stated the level of study evidence and two (e.g., Public Health Agency of Canada [76*]; NHS England [77*]) did not state either.

### Methodological quality

The AGREE-II domain scores for each of the seven guidelines are shown in Table 4 (for raw scores, see Supplementary Table 4). The mean percentage scores expressed in percentages (SD; percentage ranges) for the domains were as follows: scope and purpose 70% (SD 11.4; 67%—100%), stakeholder involvement 66% (SD 19.4; 56% -100%), rigour of development 51% (SD 33.1; 22%—100%), clarity of presentation 75% (SD 4.8; 86%—100%), applicability 63% (SD 12.4; 63%—95%) and editorial independence 36% (SD 39.8; 0%—100%).

**Table 4:**
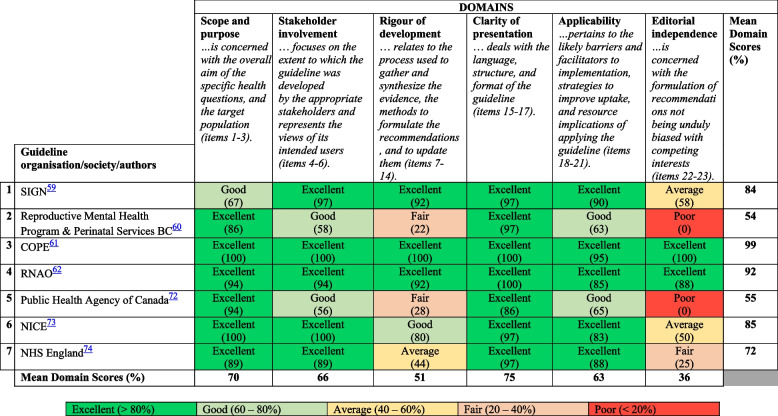
Domain scores for quality assessment according to the AGREE-II instrument

The methodological quality of the reviewed guidelines varied, with only COPE [65*] and RNAO [63*] achieving *‘excellent’* ratings (i.e., > 80%) in all six AGREE-II domains. Across guidelines, the highest AGREE-II domain scores were awarded for ‘clarity of presentation’ and the lowest were for ‘rigour of development’ and ‘editorial independence’. Unfortunately, three guidelines in this review lacked transparency about specifics of their guideline development process (e.g., search methods and/or appraisal of evidence supporting recommendations) when information was either unstated or difficult to find (SIGN [62*]; Public Health Agency Canada [76*]; NHS England [77*]). Competing interests of the guideline development group were also unacknowledged in these three guidelines and they did not provide any statement that funding bodies had no influence over guideline content. Interestingly, even relatively recent guidelines lacked transparency in terms of search methods and quality appraisal of evidence supporting recommendations (e.g., Reproductive Mental Health Program and Perinatal Services BC [75*]; Public Health Agency of Canada [76*]; NHS England [77*]).

Four (57%) guidelines (NICE [64*]; RNAO [63*]; SIGN [62*]; COPE [65*]) were assessed as ‘recommended for use’, since their quality scores were between 5 and 7, representing good quality to high quality guidelines. Three (43%) guidelines (Reproductive Mental Health Program and Perinatal Services BC [75*]; NHS England [77*]; Public Health Agency of Canada [76*]) were ‘recommended for use with modifications’, with quality scores of 4 and 5. Guidelines with excellent ratings overall shared some common features, namely clear presentation, transparency about the process of recommendation formulation and stakeholder involvement.

### Overview of each guideline

COPE [65*] published an extremely comprehensive and well considered guideline that was rated excellent overall. Many aspects of women’s identities, involving culture, ethnicity, family structure and experiences of trauma, were notably reflected upon in recommendations. An underpinning value and principle championed throughout the guideline was “perinatal mental health care should be culturally responsive and family-centred” [65*], pg.6, irrespective of setting and circumstances. The recommendations were actionable and easy to follow or navigate. The guideline ensured that their guideline addressed all domains in the AGREE-II tool [65*] and, as a result, the content was well set out, flowed seamlessly and clearly documented every facet of the guideline development process.

RNAO’s [63*] recommendations focused on perinatal depression, were easy to understand and follow, were well-evidenced and clearly stipulated, and this guideline was also rated excellent. Each recommendation had sections on values and preferences, and practice points. Values and preferences described methods to improve health equality and informed decision making for every woman, which enhanced the therapeutic richness of recommendations.

The NICE [64*] guideline included in this review was an updated version of the full guideline originally published in 2007 [81*]. The updated online version was rated excellent overall and was considerably more readable than the full downloadable guideline, which provided all technical aspects of the development process. Recommendations focused on each PMHD [82]. Cultural and trauma-informed care were also considered. However, information lacked detail when compared to the COPE [65*] and RNAO [63*] guidelines. The full NICE guideline [81]) could be improved by clearly labelling and sectioning various aspects of the recommendation and technical reports to enhance its useability.

SIGN [62*] received an overall excellent covered recommendations for a range of psychological and psychosocial assessments and/or interventions, which were clearly informed by evidence. SIGN [62*] allocated values (e.g., 1^++^, 1^+^, 2^++^, 3 etc.), which were explained in a key, to denote to the level of supporting evidence (e.g., RCTs with a very low risk of bias (1^++^) versus non-analytic studies(3)) for recommendations listed in each section. This guideline also assigned a value to indicate the quality of evidence used to formulate each recommendation, which illustrated the strength of recommendation made and increased its clinical usability.

NHS England’s [77] guide for working with partners and families affected by PMHDs was rated good overall. It covers a lot of content in remarkably succinct and visual form, which made it very accessible and easy to read. It thoughtfully considered ideas to support the wider system around mothers with PMHDs, which, as the guideline authors noted, could significantly improve outcomes for women and their families alike. Examples were provided to illustrate how clinicians can integrate these recommendations or ideas into practice.

Whilst the Public Health Agency of Canada guideline [76*] scored relatively well on the AGREE-II tool (rated average overall), the depth of content provided in its recommendations for PMHDs was minimal. The focus was on health care professionals’ assessment of PMHDs, with the aim to refer women onto specialist teams for further assessment and intervention. Nonetheless, recommendations were clearly written and actionable.

Lastly, Reproductive Mental Health Program and Perinatal Services BC [75*] received the lowest quality scores yet received an average rating overall. Despite the lower scores, it very comprehensively set out recommendations for various psychological interventions from CBT to psychodynamic psychotherapy as well as parent-infant psychotherapy. It also considered cultural aspects to delivering perinatal care and provided practice points to help mental health professionals effectively navigate this.

### Synthesis of recommendations

The synthesis of guideline recommendation themes and corresponding categories is reported in Table 5, it offers a descriptive guide to highlight areas covered well across all guidelines and areas that provided less detail. Three major psychological input themes were identified within the eligible guidelines. These were 1) therapeutic approaches in perinatal context, 2) equitable care considerations and 3) individual and systemic considerations. Key recommendations under each theme and corresponding category were compared, contrasted and summarised through narrative description. Key recommendations included across guidelines are presented in Supplementary Tables 3 and 4, which illustrate the consistency of psychosocial and psychological intervention recommendations for PMHDs.

**Table 5:** Coverage of guideline recommendation themes and associated categories for perinatal psychological mental health care

	***Antenatal Assessment***			***Antenatal Intervention***			***Postnatal Assessment***			***Postnatal Intervention***			***Total scores (xx/168)***	***% coverage***
**Theme and recommendat-ion category**	**Women**	**Mother—infant dyad**	**Partners**	**Women**	**Mother- infant dyad**	**Partners**	**Women**	**Mother- infant dyad**	**Partners**	**Women**	**Mother- infant dyad**	**Partners**		
***Therapeutic approaches in perinatal context***														
Psychosocial	11	5	4	9	3	1	11	5	6	9	3	3	**70**	**42**
Structured psychological	9	5	4	10	5	5	9	5	4	10	6	6	**78**	**46**
Focus on specific MH conditions	10	2	3	8	1	1	10	2	3	7	1	1	**49**	**29**
Trauma-informed	6	3	3	5	2	2	6	4	3	5	2	2	**43**	**26**
Use of validated questionnaires	11	1	2	3	1	0	11	1	2	3	1	0	**36**	**21**
***Equitable care considerations***														
Therapeutic relationship	5	2	2	6	2	2	5	2	2	6	2	2	**38**	**23**
Cultural/diversity	10	2	3	9	2	2	10	2	3	9	2	3	**57**	**34**
Environmental^a^	9	2	4	7	1	1	11	2	4	7	2	3	**53**	**32**
***Individual and systemic considerations***														
Recovery-oriented	4	1	0	4	1	0	4	1	0	4	3	0	**22**	**13**
Care planning	10	5	2	9	4	1	9	5	2	10	4	3	**64**	**38**
Involve significant others	6	4	1	6	3	0	6	4	1	6	5	0	**42**	**25**
Safety / Risk	9	11	1	6	5	0	9	7	1	6	5	0	**60**	**36**
***Total scores (xx/168)***	**100**	**43**	**29**	**82**	**30**	**15**	**101**	**40**	**31**	**82**	**34**	**25**		
**% coverage**	**60**	**26**	**17**	**49**	**18**	**9**	**60**	**24**	**18**	**49**	**20**	**15**		

^a^Environmental considerations—e.g., timing, location [apps / in person], frequency [of screening or sessions], clinician training / competency

#### Therapeutic approaches in perinatal context

All guidelines included some recommendations for therapeutic models of care. Structured psychological approaches (46% of coverage) were most commonly recommended across guidelines, in which 86% provided recommendations for mothers (e.g., CBT or IPT), 57% included recommendations for mother-infant dyad (e.g., parent-infant psychotherapy or video interaction guidance/VIG) and 57% for partners (e.g., CBT or couples and/or family therapy). Psychosocial approaches (42% of coverage) were also broadly considered across guidelines, in which 100% provided psychosocial recommendations for mothers, 71% for the mother-infant dyad and 43% for partners. Building women’s social connections and support network was the primary focus of psychosocial recommendations.

Five recommendations from six guidelines (SIGN [62*]; Reproductive Mental Health Program and Perinatal Services BC [75*]; COPE [65*]; RNAO [63*]; Public Health Agency Canada [76*]; NICE [64*]) related to psychosocial risk factors and mood in the context of prompt and comprehensive assessment for women. Only one guideline (NICE [64*]) noted the importance of considering any learning or intellectual disability or acquired cognitive impairments when assessing PMHDs.

Five guidelines (Reproductive Mental Health Program and Perinatal Services BC [75*]; COPE [65*]; RNAO [63*]; NHS England [77*]) recommended assessing the mother-infant/foetus relationship as an integral part of perinatal care by observing the parent-infant relationship but recommended measures varied across guidelines. For instance, COPE [65*] as well as the Reproductive Mental Health Program and Perinatal Services BC [75*] recommended using the Postpartum Bonding Questionnaire (PBQ [83]), while RNAO [63*] suggested the Edinburgh Postnatal Depression Scale (EPDS [84]) and NHS England [77*] did not specify a measure for the assessment. Two recommendations from five guidelines (SIGN [62*]; Reproductive Mental Health Program ans Perinatal Services BC [75*]; COPE [65*]; NICE [64*], 2020; NHS England [77*]) related to addressing the needs of partners, families or carers that might affect a woman with PMHDs.

Four guidelines (NICE [64*]; SIGN [62*]; RNAO [63*] and Reproductive Mental Health Program and Perinatal Services BC [75*]) offered prescriptive recommendations that were narrower in focus and were categorised based on specific PMHDs. In contrast, COPE [65*] and NHS England [77*] offered a broader approach by documenting PMHDs in generalist terms (e.g., depression / anxiety). COPE [65*] combined PMHDs into groups based on commonality and severity of mental health difficulties and offered the most comprehensive recommendations for structured psychological, psychosocial and trauma-informed care.

#### Equitable care considerations

All guidelines make recommendations on how contextual therapeutic factors should inform clinical practice. Recommendations were grouped into the following three categories: therapeutic relationship during perinatal period, cultural and diversity considerations, and environmental considerations (e.g., timing/location/frequency of therapy sessions). Four of the seven guidelines reported recommendations across all three categories.

Cultural and diversity considerations were the most comprehensively covered (34%) and specifically focused on how to ensure culturally competent, inclusive care (with 100% of guidelines providing these recommendations for mothers, 29% for the mother-infant dyad and 29% for partners). Environmental factors, such as location or timing of assessment or intervention, were equally well documented (32%): 100% of guidelines provided recommendations for mothers, 29% for the mother-infant dyad and 43% for partners. Although therapeutic relationship factors in the perinatal context (e.g., building rapport and addressing any feelings of stigma) were documented less extensively (23%), 57% provided associated recommendations for mothers, 29% for the mother-infant dyad and 29% for partners. Recommendations focused on the importance of building trust in the therapeutic relationship by ensuring therapist continuity, normalising and validating feelings, promoting a sense of collaboration, and fostering hope and optimism about therapy.

RNAO [63*], NHS England [77*] and COPE [65*] offered recommendations on equitable care factors, such as training needs for all health care professionals specific to women-centred communication, psychosocial assessment and culturally safe care. Recommendations highlighted the importance of considering how the family’s culture views PMHDs and perinatal services, and the role of men and fathers within the family. Supervision and training were recommended to enhance cultural competency by challenging the therapist’s cultural assumptions and unconscious biases particularly when working with Black and Minority Ethnic (BaME) parents, LGBTQ + parents and lone parents. SIGN [62*] suggested some equitable care recommendations but, when compared to aforementioned guidelines, the content lacked depth. SIGN [62*] noted the limited evidence-base across equitable care categories at the time of its development. Therefore, publication year could have played a role in the considerable disparity between the quality of equitable care content across guidelines.

Provision of culturally relevant information on PMHDs was recommended by five guidelines (SIGN [62*]; Reproductive Mental Health Program and Perinatal Services BC [75*]; COPE [65*]; RNAO [63*]; NICE [64*]; NHS England [77*]) along with the use of culturally appropriate cut off scores for psychometric assessment tools (COPE [65*]; RNAO [63*]). One recommendation across four guidelines (Reproductive Mental Health Program and Perinatal Services BC [75*]; COPE [65*]; RNAO [63*]; NICE [64*]) concerned facilitation of informed decision making about therapeutic interventions (i.e., informing women about the evidence-base and promote their choice over psychological interventions on offer). A further recommendation across three guidelines (COPE [65*]; RNAO [63*]; NHS England [77*]) related to the consideration of stigma and stereotypes of men’s mental health when assessing male partners.

#### Individual and systemic considerations

Six of the seven guidelines (85.7%) had recommendations pertaining to individual and systemic intervention considerations. There were four categories identified within this theme. The most common categories of recommendations related to ‘*care planning’* (38% of coverage), *‘safety and risk’* (36%) and *‘involvement of significant others’* (25%) in a woman’s perinatal psychological intervention. Few guidelines made recommendations for ‘*recovery oriented’* care (13%).

The broadest consideration of individual and systemic aspects of therapeutic intervention could be found in the Australian COPE [65*] and the English NICE [64*] guidelines (50% and 42%, respectively). Specific inclusion of systemic and individual therapeutic recommendations as per target population (i.e., mothers, mother-infant dyads and partners) varied across both of these guidelines (78% across all categories for mothers; 59% for mother-infant dyad; 0% partners within both guidelines). In contrast, SIGN [62*] and Public Health Agency of Canada [76*] were much narrower in scope (38% and 0%, respectively).

Two recommendations from six guidelines (SIGN [62*]; Reproductive Mental Health Program and Perinatal Services BC [75*]; COPE [65*]; RNAO [63*]; NICE [64*]; NHS England [77*]) concerned a woman’s support network in terms of involving and supporting significant others within her assessment and intervention. Two recommendations across four guidelines (Reproductive Mental Health Program and Perinatal Services BC [75*]; COPE [65*]; RNAO [63*]; NICE [64*]) related to risk, namely risk to self and to others with a focus on physical or emotional risk to the infant. Finally, only NHS England [77] suggested consideration of the mother's, partner's and other family members’ needs at transition points in care.

### Comparison of guideline scope

Some guidelines (SIGN [62*]; COPE [65*]; RNAO [63*]) included consensus based recommendations or practice points/notes in the absence of quality of evidence for aspects of perinatal mental health care (e.g., trauma-informed care / provision of mother-infant interventions / psychological approaches for perinatal borderline personality difficulties). Consensus based recommendations and practice points were suggested best practice based on the clinical experience of the guideline development group. Notably, there were more consensus-based recommendations and practice points than evidence-based recommendations within guidelines.

Overall, recommendations for psychological and/or psychosocial assessment and intervention with women who have PMHDs were variably covered across guidelines. Whilst the content of key recommendations was generally consistent, the level of detail and depth provided was not. For instance, no recommendation was consistently covered across every guideline. Nonetheless, structured psychological approaches were the most consistently offered in the context of antenatal and postnatal assessment and intervention. Recovery-oriented recommendations for assessment and intervention were still emerging in guidelines, potentially because the evidence base is still growing for this approach. Recommendations largely focused on women but, in more recent guidelines, it was apparent that mother-baby dyads and partners were increasingly included too.

## Discussion

This review was the first to comprehensively synthesise available clinical practice guidelines on psychosocial and psychological assessment and intervention for PMHDs with the aim to summarise and explore the consistency of key recommendations. The scope and breadth of guidelines varied greatly as did their methodological quality, with only two guidelines achieving high ratings (QA score > 90%), namely COPE [65*] and RNAO [63*]. As psychological intervention outcomes are influenced by various therapeutic factors, such as multi-cultural and therapeutic competence [85–87] and alliance [88], these factors were considered across many guidelines but were most thoroughly documented within those highly rated (e.g., COPE [65*]; RNAO [63*]).

Guidelines typically focused on PMHDs more broadly. COPE [65*], NICE [64*], Public Health Agency of Canada [76*] and NHS England [77*] did not itemise recommendations based on specific perinatal mental health diagnoses. Instead, COPE [65] combined recommendations for assessment and treatment approaches with perinatal anxiety and depression, and differentiated these from recommendations for severe PMHDs (e.g., borderline personality difficulties/psychosis). Given the high rate of comorbidity in the perinatal population [89], COPE [65*], NICE [64*] and NHS England [77*] helpfully attempted to focus the clinician on meeting the needs of the service-user in the context of their PMHD rather than specifically focusing on their PMHD. It was encouraging that the mother-infant dyad was acknowledged and that partners were considered in recommendations, too. However, this was not consistently reflected across all guidelines. Public Health Agency of Canada [76*] only focused on women with no consideration for partners or the mother-infant relationship.

Some recommendations were universally agreed upon by most guideline development groups (SIGN [62*]; Reproductive Mental Health Program and Perinatal Services BC [75*]; COPE [65*]; RNAO [63*]; NICE [64*]). For instance, the usefulness of CBT and IPT for PMHDs was highlighted along with the importance of discussing treatment options and/or timing to ensure women can make informed decisions about their potential engagement with therapy. Working with the woman’s significant other(s) in the assessment and intervention phases was strongly recommended to address any relationship difficulties or unhelpful communication patterns that could be impacting her mental health (Reproductive Mental Health Program and Perinatal Services BC [75*]; COPE [65*]; RNAO [63*]; NICE [64*]).

Many guidelines expressed the importance of assessing mother-baby interactions as an fundamental part of postnatal care due to the possible impact PMHDs can have on the mother-baby relationship (Reproductive Mental Health Program and Perinatal Services BC [75*]; COPE [65*]; RNAO [63*]; NICE [64*]). Offering additional interventions, specifically directed at the mother-baby relationship, was recommended if impairment was evident. Acknowledging the woman's role in caring for her baby was noted in COPE [65*] and NICE [64*] along with the importance of mental health professionals supporting women to care for their baby in a non-judgmental and compassionate way.

Whilst recommendations on (mostly male) partners’ mental health were not formalised in almost all of the guidelines, practice points were offered in some to encourage exploration of stigma and stereotypes when assessing a partner’s mental health (COPE [65*]; RNAO [63*]). Most recommendations focused on the potential role of partners in supporting a woman’s psychological therapy rather than on specific intervention for the partner’s mental health (SIGN [62*]; Reproductive Mental Health Program and Perinatal Services BC [75*]; RNAO [63*]). COPE [65*] tentatively offered suggestions on psychological interventions for fathers, which were notably based on few research papers and involved adopting novel ways to engage, assess and support fathers (e.g., through mobile technology). There is great need for all guidelines to consider recommendations on working with familial systems, including non-gestational parents of both sexes, around women with PMHDs [90] to improve familial mental health, relationships and bonding with the infant.

The majority of lifetime costs caused by PMHDs relate to adverse impacts on children [91]. The time from conception to two years old is a unique developmental period for infants that sets the foundations of lifelong emotional and physical wellbeing [92]. The mother’s ability to be sensitive, engaged and responsive (or mind-mindedness) with her infant in the first years of life is considered important for enhancing the quality of early mother-infant interaction, which can improve the security of the mother-infant bond and eventual attachment (see Meins [93]; Crittenden [94]). Perinatal mental health services have an equal responsibility for the infant to improve the infant’s care, development and safety [95]. Recommendations for the mother-infant relationship in perinatal clinical practice guidelines play a fundamental role in improving therapeutic practice and long-term outcomes for the infant [96] with an aim to reduce costs to society over their lifetime.

NICE [64*] stressed the importance of therapists facilitating manualised therapies. However, this was not indicated across other guidelines, which advocated a less restrictive approach. For example, it was strongly recommended that therapies were delivered by adequately trained professionals (SIGN [62*]; Reproductive Mental Health Program and Perinatal Services BC [75*]; COPE [65*]; NHS England [77*]). NICE [64*] did not recommend specific manualised therapies, which is likely an area for further exploration due to the lack of manualised evidence-based therapy modalities in the perinatal period. Clinical psychologists commonly advocate tailored and formulation driven interventions to meet the highly specialised needs of women in the perinatal period [24]. Fonagy and Luyten [97] further argued that the most important aspects of psychological interventions are experience and flexibility of therapists. Most guidelines also noted the fundamental importance of clinical supervision to ensure good clinical practice in the facilitation of psychological interventions (SIGN [62*]; COPE [65*]; NICE [64*]). However, no guideline indicated specifics about the regularity of clinical supervision.

Cultural aspects of a woman’s care in the perinatal period were carefully considered and integrated into some guidelines (COPE [65*]; RNAO [63*]; NICE [64*]; NHS England [77*]). Considering culturally relevant cut off scores for the Edinburgh Postnatal Depression Scale [84] or the cultural appropriateness of psychosocial assessment tools were recommended (Reproductive Mental Health Program ans Perinatal Services BC [75*]; COPE [65*]; RNAO [63*]). Understanding how cultural background may influence a woman or significant other’s perception of PMHDs and the role men have in different cultures was encouraged. Training in women-centred communication and culturally safe care was strongly proposed for all health care professionals to facilitate therapeutic relationships with women and their families during the perinatal period.

Transparency about the guideline development process can fundamentally underpin the trustworthiness of guidelines and was an issue for some guidelines included in this review. Ford et al. [98] indicated a need for continuous review of the evidence-base following any explosive growth as it may impact the type of evidence used to formulate guideline recommendations, which could lead to changes in policy. In this review, some guideline development groups provided few specifics about their development process that would have enhanced its transparency and trustworthiness (SIGN [62*]; Public Health Agency Canada [76*]; NHS England [77*]).

Consensus-based recommendations provided within guidelines (SIGN [62*]; COPE [65*]; RNAO [63*]) held significant plausibility for implementation within perinatal mental health care. However, the method by which these recommendations were formulated, highlighted a need to build on the evidence base for the associated aspects of perinatal mental health care. For example, to further research the benefits of trauma informed care and provision of mother-infant interventions in perinatal interventions. Further development of this evidence base has the potential to add weight and consistency to recommendations provided, enhance the implementation of clinical practice guidelines within perinatal mental health settings and therefore improve long-term outcomes for women and their families.

### Limitations of this review

Firstly, only clinical practice guidelines published in English were eligible for inclusion. Those written in other languages may exist, for example, we excluded one record that was non-English and which may have been relevant to this review. This non-English guideline focused on structuring perinatal care, which could have added an additional cultural stance to this review along with how perinatal psychological care is delivered in Germany. Translation for the German guideline was not sought due to time and resource constraints.

Secondly, items on the AGREE-II tool were assigned low scores in the absence of necessary information to evidence whether criteria were met across the various AGREE-II domains (e.g., rigour of development). It is recognised that guideline development groups could have undertaken required processes during guideline development as outlined by the AGREE-II criteria, but this was not captured in their published documents. In addition, the AGREE-II primarily focuses on methodological quality and internal validity of guidelines and to a lesser extent the external validity of the recommendations [99].

Thirdly, we set out to find the most widely used guidance across all countries and were aware state-wide guidelines for perinatal mental health likely existed in the US, but these were not found in our searches. The only US guidance retrieved through International Guidelines Network and Guideline Central databases were related to age and perinatal health.

Finally, this review focused on maternal mental health. There may have been guidelines for infant mental health that focus on the mother-infant relationship and paternal mental health, which were not included in this review.

### Conclusions

Seven clinical practice guidelines for psychological and psychosocial assessment and intervention in the perinatal period that met our inclusion criteria exist and there is considerable variability in guideline quality, breadth and depth of recommendations. Clinicians need to be aware of the quality disparities across guidelines and may need to look beyond their local guidelines to improve the level of clinical care using the highest quality guidelines for psychological and psychosocial assessment and intervention indications for PMHDs.

It is reassuring to see both women’s perinatal psychological and/or psychosocial intervention being situated within a wider familial system and the emerging inclusion of partners’ mental health across guidelines. However, there is a need to continue building the evidence for partners’ mental health to ensure development of high quality therapeutic interventions. More significant efforts should be made to improve reporting of systemically informed recommendations to reinforce the benefits of social support for women. This process could involve how to include partners and families within a woman’s psychological intervention.

COPE [65*] was exemplary of a ‘gold standard’ guideline; it was a well evidenced, exceptionally informative, easy to navigate, transparent and a thoughtfully documented guideline for PMHDs. However, there is a need to improve the quality of evidence underpinning guidelines and the rigour of their development along with increased transparency about the guideline development process in line with the AGREE-II tool domains. Improved transparency and evidence used for recommendation formation could influence guideline implementation and, ultimately, enhance the psychological assessment and interventions for women, the mother-baby dyad and partners during the perinatal period. 

### Supplementary Information

**Additional file 1: Supplementary Table 1.** Guideline recommendation themes and associated subcategories for psychological assessment and intervention in perinatal period.

**Additional file 2: Supplementary Table 2.** Comparison of key recommendations for psychosocial and psychological assessment in the perinatal period .

**Additional file 3: Supplementary Table 3.** Comparison of recommendations for psychosocial and psychological intervention in the perinatal period.

**Additional file 4: Supplementary Table 4.** Raw scores for the AGREE-II tool across each of the seven guidelines.

## Data Availability

All data analysed during this study are included in this published article and its supplementary information files.

## Abbreviations

AGREE II: Appraisal of Guidelines for Research & Evaluation Instrument
AW: Anja Wittkowski
AR: Amy Rathbone
BC: British Columbia
CBT: Cognitive Behavioural Therapy
COPE: Centre of Perinatal Excellence
IPT: Interpersonal Psychotherapy
JOB: Jayne O’Brien
LG: Lynsey Gregg
PMHD: Perinatal mental health difficulties
PRIME-RU: Perinatal Mental Health and Parenting Research Unit
NICE: National Institute of Clinical Excellence
NHS: National Health Service
RNAO: Registered Nursing Association of Ontario
SIGN: Scottish Intercollegiate Guidelines Network
UK: United Kingdom
US: United States (of America)
ZK: Zaynab Kahn
