# Supplementary material for: A systematic review of clinical psychological guidance for perinatal mental health
Source: BMC Psychiatry. 2023 Oct 30;23:790. doi: 10.1186/s12888-023-05173-1 (PMC10614401; doi:10.1186/s12888-023-05173-1)
Supplement: Supplementary file 1 — Additional file 1: Supplementary Table 1. Guideline recommendation themes and associated subcategories for psychological assessment and intervention in perinatal period. [file 12888_2023_5173_MOESM1_ESM.pdf]

Supplementary Table 1: Guideline recommendation themes and associated subcategories for psychological assessment and intervention in perinatal period.

No.	Theme and recommendation category	*Antenatal Assessment*			*Antenatal Intervention*			*Postnatal Assessment*			*Postnatal Intervention*			*% coverage*
		**Women**	**Mother -infant dyad**	**Partners**	**Women**	**Mother - infant dyad**	**Partners**	**Women**	**Mother - infant dyad**	**Partners**	**Women**	**Mother - infant dyad**	**Partners**	
**1**	***Therapeutic approaches in perinatal context***													
	Psychosocial	✓✓	✓	-	✓	-	-	✓✓	-	-	✓	-	-	**29**
	Structured psychological	✓	-	-	✓	-	-	✓	-	-	✓	✓	✓	**25**
	Focus on specific MH conditions	✓✓	-	-	✓✓	-	-	✓✓	-	-	✓	-	-	**29**
	Trauma-informed	-	-	-	-	-	-	-	-	-	-	-	-	**0**
	Use of validated questionnaires	✓✓	-	-	-	-	-	✓✓	-	-	-	-	-	**17**
	Equitable care considerations													
	Therapeutic relationship	-	-	-	✓	-	-	-	-	-	✓	-	-	**8**
	Cultural/diversity	✓	-	-	✓	-	-	✓	-	-	✓	-	-	**17**
	Environmental **	✓	-	-	✓	-	-	✓	-	-	✓	-	-	**17**
	Individual and systemic considerations													
	Recovery-oriented	-	-	-	-	-	-	-	-	-	-	-	-	**0**
	Care planning	✓	-	-	✓	-	-	✓	-	-	✓	-	-	**17**
	Involve significant others	-	-	-	-	-	-	-	-	-	-	-	✓	**4**
	Safety / Risk	✓	-	-	✓	-	-	✓	-	-	✓	-	-	**17**
**2**	***Therapeutic approaches in perinatal context***													
	Psychosocial	✓✓	✓	✓	✓✓	-	-	✓✓	✓✓	✓✓	✓✓	-	-	**58**
	Structured psychological	✓	-	✓	✓✓	✓✓	✓✓	✓	-	✓	✓✓	✓✓	✓✓	**67**

		Antenatal Assessment			*Antenatal Intervention*			*Postnatal Assessment*			*Postnatal Intervention*			*% coverage*
**No.**	**Theme and recommendation category**	**Women**	**Mother -infant dyad**	**Partners**	**Women**	**Mother - infant dyad**	**Partners**	**Women**	**Mother - infant dyad**	**Partners**	**Women**	**Mother - infant dyad**	**Partners**	
	Focus on specific MH conditions	✓✓	✓	✓✓	✓	-	✓	✓✓	✓	✓✓	✓	-	✓	**58**
	Trauma-informed	-	-	-	-	-	-	-	-	-	-	-	-	**0**
	Use of validated questionnaires	✓✓	-	✓	-	-	-	✓✓	-	✓	-	-	-	**25**
	Equitable care considerations													
	Therapeutic relationship	-	-	-	-	-	-	-	-	-	-	-	-	**0**
	Cultural/diversity	✓	-	-	-	-	-	✓	-	-	-	-	-	**8**
	Environmental **	✓✓	-	✓	-	-	-	✓✓	-	✓	-	-	-	**25**
	Individual and systemic considerations													
	Recovery-oriented	-	-	-	-	-	-	-	-	-	-	-	-	**0**
	Care planning	✓	-	-	✓	✓	✓	-	-	-	✓✓	✓	✓	**33**
	Involve significant others	-	-	-	-	-	-	-	-	-	-	-	-	**0**
	Safety / Risk	✓✓	✓✓	-	✓✓	✓✓	-	✓✓	✓✓	-	✓✓	✓✓	-	**67**
**3**	***Therapeutic approaches in perinatal context***													
	Psychosocial	✓✓	✓	✓	✓✓	✓	✓	✓✓	✓	✓✓	✓✓	✓	✓	**71**
	Structured psychological	✓✓	✓✓	-	✓✓	✓	✓	✓✓	✓✓	-	✓✓	✓	✓	**67**
	Focus on specific MH conditions	✓✓	✓	✓	✓✓	✓	-	✓✓	✓	✓	✓✓	✓	-	**58**
	Trauma-informed	✓✓	✓	✓	✓✓	✓	✓	✓✓	✓	✓	✓✓	✓	✓	**67**
	Use of validated questionnaires	✓✓	✓	✓	✓	✓	-	✓✓	✓	✓	✓	✓	-	**50**

No.	Theme and recommendation category	*Antenatal Assessment* Women	*Antenatal Assessment* Mother-infant dyad	*Antenatal Assessment* Partners	*Antenatal Intervention* Women	*Antenatal Intervention* Mother - infant dyad	*Antenatal Intervention* Partners	*Postnatal Assessment* Women	*Postnatal Assessment* Mother - infant dyad	*Postnatal Assessment* Partners	*Postnatal Intervention* Women	*Postnatal Intervention* Mother - infant dyad	*Postnatal Intervention* Partners	*% coverage*
	Equitable care considerations													
	Therapeutic relationship	✓✓	✓	✓	✓✓	✓	✓	✓✓	✓	✓	✓✓	✓	✓	**67**
	Cultural/diversity	✓✓	✓	✓✓	✓✓	✓	✓✓	✓✓	✓	✓✓	✓✓	✓	✓✓	**83**
	Environmental **	✓✓	✓	✓	✓	-	✓ ***	✓✓	✓	✓	✓	✓	✓ ***	**54**
	Individual and systemic considerations													
	Recovery-oriented	✓✓	✓	-	✓✓	✓	-	✓✓	✓	-	✓✓	✓	-	**50**
	Care planning	✓✓	✓	-	✓✓	✓	-	✓✓	✓	-	✓✓	✓	-	**50**
	Involve significant others	✓	✓	-	✓	✓	-	✓	✓	-	✓	✓	-	**33**
	Safety / Risk	✓✓	✓✓	-	✓✓	✓✓	-	✓✓	✓✓	-	✓✓	✓✓	-	**67**
**4**	***Therapeutic approaches in perinatal context***													
	Psychosocial	✓	-	-	✓✓	-	-	✓	-	-	✓✓	-	-	**25**
	Structured psychological	✓✓	-	-	✓✓	-	-	✓✓	-	-	✓✓	-	-	**33**
	Focus on specific MH conditions	✓	-	-	✓	-	-	✓	-	-	✓	-	-	**17**
	Trauma-informed	✓✓	-	-	✓	-	-	✓✓	-	-	✓	-	-	**25**
	Use of validated questionnaires	✓	-	-	-	-	-	✓	-	-	-	-	-	**8**
	Equitable care considerations													
	Therapeutic relationship	✓✓	-	-	✓✓	-	-	✓✓	-	-	✓✓	-	-	**33**
	Cultural/diversity	✓✓	-	-	✓✓	-	-	✓✓	-	-	✓✓	-	-	**33**
	Environmental **	✓✓	-	-	✓✓	-	-	✓✓	-	-	✓✓	-	-	**33**

No.	Theme and recommendation category	*Antenatal Assessment*			*Antenatal Intervention*			*Postnatal Assessment*			*Postnatal Intervention*			*% coverage*
		**Women**	**Mother -infant dyad**	**Partners**	**Women**	**Mother - infant dyad**	**Partners**	**Women**	**Mother - infant dyad**	**Partners**	**Women**	**Mother - infant dyad**	**Partners**	
	Individual and systemic considerations													
	Recovery-oriented	✓	-	-	✓	-	-	✓	-	-	✓	-	-	**17**
	Care planning	✓✓	-	-	✓✓	-	-	✓✓	-	-	✓✓	-	-	**33**
	Involve significant others	✓	-	-	✓✓	-	-	✓	-	-	✓✓	-	-	**25**
	Safety / Risk	✓	-	-	-	-	-	✓	-	-	-	-	-	**8**
**5**	*Therapeutic approaches in perinatal context*													
	Psychosocial	✓✓	-	-	-	-	-	✓✓	-	-	-	-	-	**17**
	Structured psychological	-	-	-	-	-	-	-	-	-	-	-	-	**0**
	Focus on specific MH conditions	✓	-	-	-	-	-	✓	-	-	-	-	-	**8**
	Trauma-informed	-	-	-	-	-	-	-	-	-	-	-	-	**0**
	Use of validated questionnaires	✓✓	-	-	✓	-	-	✓✓	-	-	✓	-	-	**25**
	Equitable care considerations													
	Therapeutic relationship	-	-	-	-	-	-	-	-	-	-	-	-	**0**
	Cultural/diversity	✓	-	-	✓	-	-	✓	-	-	✓	-	-	**17**
	Environmental **	✓	-	-	-	-	-	✓	-	-	-	-	-	**8**
	Individual and systemic considerations													
	Recovery-oriented	-	-	-	-	-	-	-	-	-	-	-	-	**0**
	Care planning	-	-	-	-	-	-	-	-	-	-	-	-	**0**
	Involve significant others	-	-	-	-	-	-	-	-	-	-	-	-	**0**
	Safety / Risk	-	-	-	-	-	-	-	-	-	-	-	-	**0**

No.	Theme and recommendation category	*Antenatal Assessment*			*Antenatal Intervention*			*Postnatal Assessment*			*Postnatal Intervention*			*% coverage*
		**Women**	**Mother -infant dyad**	**Partners**	**Women**	**Mother - infant dyad**	**Partners**	**Women**	**Mother - infant dyad**	**Partners**	**Women**	**Mother - infant dyad**	**Partners**	
**6**	***Therapeutic approaches in perinatal context***													
	Psychosocial	✓	✓	-	✓	✓	-	✓	✓	-	✓	✓	-	**33**
	Structured psychological	✓	✓	✓	✓✓	✓	✓✓	✓	✓	✓	✓✓	✓	✓	**63**
	Focus on specific MH conditions	✓✓	-	-	✓✓	-	-	✓✓	-	-	✓✓	-	-	**33**
	Trauma-informed	✓	✓	✓	✓	✓	✓	✓	✓✓	✓	✓	✓	✓	**54**
	Use of validated questionnaires	✓✓	-	-	✓	-	-	✓✓	-	-	✓	-	-	**25**
	Equitable care considerations													
	Therapeutic relationship	✓	✓	✓	✓	✓	✓	✓	✓	✓	✓	✓	✓	**50**
	Cultural/diversity	✓	-	-	✓	-	-	✓	-	-	✓	-	-	**17**
	Environmental **	✓	-	-	✓	-	-	✓	-	-	✓	-	-	**17**
	Individual and systemic considerations													
	Recovery-oriented	✓	-	-	✓	-	-	✓	-	-	✓	-	-	**17**
	Care planning	✓✓	✓✓	-	✓	✓	-	✓✓	✓✓	-	✓	✓	-	**50**
	Involve significant others	✓✓	✓✓	-	✓	✓	-	✓✓	✓✓	-	✓	✓	-	**50**
	Safety / Risk	✓✓	✓✓	-	✓	✓	-	✓✓	✓✓	-	✓	✓	-	**50**
**7**	***Therapeutic approaches in perinatal context***													
	Psychosocial	✓	✓	✓✓	✓	✓	✓✓	✓	✓	✓✓	✓	✓	✓✓	**16**
	Structured psychological	✓✓	✓✓	✓✓	✓	✓	✓	✓✓	✓✓	✓✓	✓	✓	✓	**18**
	Focus on specific MH conditions	-	-	-	-	-	-	-	-	-	-	-	-	**0**
	Trauma-informed	✓	✓	✓	✓	-	-	✓	✓	✓	✓	-	-	**8**

		Antenatal Assessment			*Antenatal Intervention*			*Postnatal Assessment*			*Postnatal Intervention*			
**No.**	**Theme and recommendation category**	**Women**	**Mother -infant dyad**	**Partners**	**Women**	**Mother - infant dyad**	**Partners**	**Women**	**Mother - infant dyad**	**Partners**	**Women**	**Mother - infant dyad**	**Partners**	***% coverage***
	Use of validated questionnaires	-	-	-	-	-	-	-	-	-	-	-	-	**0**
	Equitable care considerations													
	Therapeutic relationship	-	-	-	-	-	-	-	-	-	-	-	-	**0**
	Cultural/diversity	✓✓	✓	✓	✓✓	✓	✓	✓✓	✓	✓	✓✓	✓	✓	**16**
	Environmental **	✓✓	✓	✓✓	✓✓	✓	✓✓	✓✓	✓	✓✓	✓✓	✓	✓✓	**20**
	Individual and systemic considerations													
	Recovery-oriented	-	-	-	-	-	-	-	-	-	-	-	-	**0**
	Care planning	✓✓	✓✓	✓✓	✓✓	✓	✓✓	✓✓	✓✓	✓✓	✓✓	✓	✓✓	**22**
	Involve significant others	✓✓	✓	✓	✓✓	✓	✓	✓✓	✓	✓	✓✓	✓	✓	**16**
	Safety / Risk	✓	✓	✓	-	-	✓	✓	✓	✓	-	-	✓	**8**
**Total % of coverage** (rounded to nearest whole number)		**60**	**26**	**17**	**49**	**18**	**9**	**60**	**24**	**18**	**49**	**20**	**15**	

---

1 – SIGN[48]
2 – Reproductive Mental Health Program & Perinatal Services BC[60]
3 – COPE[51]
4 – RNAO[49]
5 – Public Health Agency Canada[61]
6 – NICE[50]
7 – NHS England[62]
