# Supplementary material for: A systematic review of clinical psychological guidance for perinatal mental health
Source: BMC Psychiatry. 2023 Oct 30;23:790. doi: 10.1186/s12888-023-05173-1 (PMC10614401; doi:10.1186/s12888-023-05173-1)
Supplement: Supplementary file 2 — Additional file 2: Supplementary Table 2. Comparison of key recommendations for psychosocial and psychological assessment in the perinatal period . [file 12888_2023_5173_MOESM2_ESM.pdf]

Supplementary Table 2: Comparison of key recommendations for psychosocial and psychological assessment in the perinatal period

	Antenatal							Postnatal						
	**1**	**2**	**3**	**4**	**5**	**6**	**7**	**1**	**2**	**3**	**4**	**5**	**6**	**7**
**Women**														
Therapeutic approaches in perinatal context														
Assess psychosocial risk factors as early as practical in pregnancy and again after the birth.	❋	❋	❋	❋	❋	❋	-	❋	❋	❋	❋	❋	❋	-
If using a tool to assess psychosocial risk, administer the ANRQ during pregnancy.	-	-	❋	❋	❋	❋	-	-	-	-	-	-	-	-
Psychosocial assessment in the antenatal period for the purposes of identifying risk of postnatal depression should not be routinely offered.	❋	-	-	-	-	-	-	-	-	-	-	-	-	-
Undertake psychosocial assessment in conjunction with a tool that screens for current symptoms of depression/anxiety (i.e. the EPDS).	-	-	❋	-	❋	-	-	-	-	❋	-	❋	-	-
Complete the first antenatal screening as early as practical in pregnancy and repeat screening at least once later in pregnancy.	❋	❋	❋	❋	-	❋	-	-	-	-	-	-	-	-
Complete the first postnatal screening 6–12 weeks after birth and repeat screening at least once in the first postnatal year.	-	-	-	-	-	-	-	❋	❋	❋	❋	-	❋	-
Conduct or facilitate access to a comprehensive perinatal depression assessment with persons who screen positive for depression (i.e. EPDS score of 13 or more).	❋	❋	❋	❋	❋	❋	-	❋	❋	❋	❋	❋	❋	-
Promote early identification and treatment of anxiety conditions in perinatal women by enquiring about risk factors (e.g., personal and/or family history	-	❋	❋	-	❋	-	-	-	❋	❋	-	❋	-	-

Key recommendations extracted from the following:
[1] SIGN[48]
[2] Reproductive Mental Health Program & Perinatal Services BC[60]
[3] COPE[51]
[4] RNAO[49]
[5] Public Health Agency of Canada[61]
[6] NICE[50]
[7] NHS England[62]

	Antenatal							Postnatal						
	1	2	3	4	5	6	7	1	2	3	4	5	6	7
of anxiety) and/or direct observation (e.g., excessive concerns about foetus/pregnancy or baby)														
Promote early identification and treatment of bipolar disorder in perinatal women by enquiring about risk factors (personal or family history of bipolar disorder and/or postpartum psychosis) and/or direct observation or reports (e.g., unusual behaviour, racing thoughts, distractible, inflated self-esteem or grandiosity, disorganized thoughts, erratic and impulsive behaviour, rapid speech, difficulty sleeping).	-	✵	✵	-	-	-	-	-	✵	✵	-	-	-	-
Equitable care considerations														
All health professionals providing care in the perinatal period should receive training in woman-centred communication skills, psychosocial assessment and culturally safe care.	-	-	✵	✵	-	-	✵	-	-	✵	✵	✵		✵
Recognise that women who have a mental health problem (or are worried that they might have) may be unwilling to disclose or discuss their problem because of fear of stigma, negative perceptions of them as a mother or fear that their baby might be taken into care			✵		✵	✵	-			✵		✵	✵	-
Consider language and cultural appropriateness of any tool used to assess psychosocial risk.	-	-	✵	✵	-	-	-	-	-	✵	✵	-	-	-
Use appropriately translated versions of the EPDS with culturally relevant cut-off scores. Consider language and cultural appropriateness of the tool.	-	✵	✵	✵	-	-	-	-	✵	✵	✵	-	-	-
When assessing or treating a mental health problem in pregnancy or the postnatal period, take account of any learning disabilities or acquired cognitive impairments, and assess the need to consult with a specialist when developing care plans.	-	-	-	-	-	✵	-	-	-	-	-	-	✵	-
Individual and systemic considerations														
Carry out a risk assessment in conjunction with the woman and, if she agrees, her partner, family or carer. Focus on areas that are likely to present possible risk such as self-neglect, self-harm, suicidal thoughts and intent, risks to	-	✵	✵	-	-	✵	✵	-	✵	✵	-	-	✵	✵

	Antenatal							Postnatal						
	1	2	3	4	5	6	7	1	2	3	4	5	6	7
others (including the baby), smoking, drug or alcohol misuse and domestic violence and abuse.														
Explore who is in the mother’s family and support network, at the earliest opportunity.	✵	✵	✵	-	-	✵	✵	✵	✵	✵	-	-	✵	✵
If a woman agrees, provide information to and involve her significant other(s) in discussions and assessments about her emotional wellbeing and mental health throughout the perinatal period.	✵	✵	✵	✵	-	✵	-	✵	✵	✵	✵	-	✵	-
Regularly ask questions about the family system throughout the mother’s involvement with the service, as key relationships and wishes may change.	-	-	-	-	-	-	✵	-	-	-	-	-	-	✵
**Mother-baby dyad**														
Therapeutic approaches in a perinatal context														
Discuss treatment and prevention options and any particular concerns the woman has about the pregnancy or the foetus or baby. Provide information to the woman and, if she agrees, her partner, family or carer, about the potential benefits of psychological interventions.	-	-	✵	✵	-	✵	✵		-	✵	✵	-	✵	✵
Assessment and diagnosis of a suspected mental health problem in pregnancy and the postnatal period should include the woman's experience of pregnancy and any problems experienced by her, the foetus or the baby, and the mother-baby relationship.	-	✵	✵	-	-	✵	-	-	✵	✵	-	-	✵	-
Assess the mother-infant interaction as an integral part of postnatal care and refer to a parent-infant therapist as available and appropriate.	-	-	-	-	-	-	-	-	✵	✵	✵	-	✵	-
Recognise that some women with a mental health problem may experience difficulties with the mother-baby relationship. Assess the nature of this relationship, including verbal interaction, emotional sensitivity and physical care, at all postnatal contacts. Discuss concerns the woman has about her relationship with her baby and provide information and treatment for the mental health problem.	-	-	-	-	-	-	-	-	✵	✵	✵	-	✵	-

	Antenatal							Postnatal						
	1	2	3	4	5	6	7	1	2	3	4	5	6	7
Promote early identification and treatment of anxiety disorders in perinatal women by direct observation (e.g. excessive concerns about foetus/pregnancy or baby).	-	✱	✱	-	-	✱	-	-	✱	✱	-	-	✱	-
Equitable care considerations														
Take into account and, if appropriate, assess and address the needs of partners, families and carers that might affect a woman with a mental health problem in pregnancy and the postnatal period. Including the welfare of the baby and other dependent children.	-	✱	✱	-	-	✱	✱	-	✱	✱	-	-	✱	✱
Seek guidance/support from bicultural health workers when assessing mother-infant interaction in migrant and refugee women, to ensure that assessment is not informed by unconscious bias.	-	-	✱	-	-	-	-	-	-	✱	-	-	-	-
Acknowledge the woman's role in caring for her baby and support her to do this in a non-judgmental and compassionate way.	-	-	✱	-	-	✱	-	-	-	✱	-	-	✱	-
Individual and systemic considerations														
Involve the woman and, if she agrees, her partner, family or carer, in all decisions about her care and the care of her baby.	-	-	✱	✱	-	✱	✱	-	-	✱	✱	-	✱	✱
Carry out a risk assessment focus on risks to others (including the baby)	-	✱	✱	✱	-	✱	-	-	✱	✱	✱	-	✱	-
Assess the risk of harm to the infant if significant difficulties are observed with the mother-infant interaction, the woman discloses that she is having thoughts of harming her infant and/or there is concern about the mother's mental health.	-	✱	✱	✱	-	✱	-	-	✱	✱	✱	-	✱	-
**Partners**														
Therapeutic approaches in perinatal context														
Take into account and, if appropriate, assess and address the needs of partners, families and carers that might affect a woman with a mental health problem in pregnancy and the postnatal period. Including, the role of the partner, family or carer in providing support, and the potential effect of any mental health problem on the woman's relationship with her partner, family or carer.	✱	✱	✱	-	-	✱	✱	✱	✱	✱	-	-	✱	✱

	Antenatal							Postnatal						
	1	2	3	4	5	6	7	1	2	3	4	5	6	7
Be aware of the father/partner’s mental health and how this affects the mother and baby, and how the pregnancy affects the father/partner and other family members’ mental health and wellbeing.	✵	✵	✵	-	-	✵	✵	✵	✵	✵	-	-	✵	✵
Consider ways in which they can involve family members in the mother’s care and support family members as individuals, as partners/relatives to the mother, as parents/relatives to the baby.	✵	-	✵	✵	-	✵	✵	✵	-	✵	✵	-	✵	✵
Equitable care considerations														
Where appropriate, consider stigma and stereotypes in men’s mental health when assessing partners.	-	-	✵	-	-	-	✵	-	-	✵	-	-	-	✵
Be aware of cultural considerations, such as how the family’s culture views perinatal mental health disorders and services, and the role of men and fathers within the family	-	-	✵	✵	-	-	✵	-	-	✵	✵	-	-	✵
Individual and systemic considerations														
Consider partners' and other family members' needs at transition points alongside the needs of the mother.	-	-	-	-	-	-	✵	-	-	-	-	-	-	✵
