# Supplementary material for: A systematic review of clinical psychological guidance for perinatal mental health
Source: BMC Psychiatry. 2023 Oct 30;23:790. doi: 10.1186/s12888-023-05173-1 (PMC10614401; doi:10.1186/s12888-023-05173-1)
Supplement: Supplementary file 3 — Additional file 3: Supplementary Table 3. Comparison of recommendations for psychosocial and psychological intervention in the perinatal period. [file 12888_2023_5173_MOESM3_ESM.pdf]

Supplementary Table 3: Comparison of recommendations for psychosocial and psychological intervention in the perinatal period

	Antenatal							Postnatal						
	1	2	3	4	5	6	7	1	2	3	4	5	6	7
**Women**														
Therapeutic approaches in perinatal context														
Provide structured psychoeducation to women with symptoms of depression in the perinatal period.	-	✵	✵	✵	-	-	-	-	✵	✵	✵	-	-	-
Advise women with symptoms of depression in the postnatal period of the potential benefits of a social support group and/or seeking support from partners/family members/peers.	✵	✵	✵	✵	-	✵	-	✵	✵	✵	✵	-	✵	-
Promote self-care strategies for persons at risk for or experiencing perinatal mental health difficulties including:<br>1. Time for self<br>2. Exercise<br>3. Relaxation<br>4. Sleep	✵	✵	✵	✵	-	-	-	✵	✵	✵	✵	-	-	-
Provide or facilitate access to professionally-led psychosocial interventions, including non-directive counselling, for persons with perinatal depression.	-	-	-	✵	-	-	-	-	-	-	✵	-	-	-
Advise women with depression or anxiety disorder in the postnatal period of the possible benefits of directive counselling.	-	-	✵	-	-	-	-	-	-	✵	-	-	-	-
Provide and facilitate access to individual structured psychological interventions, such as cognitive behavioural therapy, psychodynamic psychotherapy or	✵	✵	✵	✵	-	✵	-	✵	✵	✵	✵	-	✵	-

1 SIGN[48]
2 Reproductive Mental Health Program & Perinatal Services BC[60]
3 COPE[51]
4 RNAO[49]
5 Public Health Agency of Canada[61]
6 NICE[50]
7 NHS England[62]

	Antenatal							Postnatal						
	1	2	3	4	5	6	7	1	2	3	4	5	6	7
interpersonal psychotherapy, for women with mild to moderate anxiety and/or depression in the perinatal period.														
For women with moderate to severe depression or anxiety in perinatal period, psychological therapies can be a useful adjunct, usually once medications have become effective.	-	✵	✵	✵	-	✵	-	-	✵	✵	✵	-	✵	-
Where possible and appropriate, provide women with borderline personality difficulties with structured psychological therapies (e.g. DBT) that are specifically designed for this condition and conducted by adequately trained and supervised health professionals.	-	-	✵	-	-	-	-	-	-	✵	-	-	-	-
For women with borderline personality disorder who have often experienced complex trauma, trauma-informed care and specific support for health professionals in dealing with challenging behaviours is a priority.	-	-	✵	-	-	-	-	-	-	✵	-	-	-	-
CBT, IPT and behavioural couples therapy for bipolar depression	-	✵	-	-	-	✵	-	-	✵	-	-	-	✵	-
Structured individual, group and family interventions designed for bipolar disorder to reduce the risk of relapse.	-	✵	-	-	-	✵	-	-	✵	-	-	-	✵	-
Psychological interventions (CBT or family intervention) for a woman with psychosis or schizophrenia who becomes pregnant and is at risk of relapse arising from stress associated with pregnancy or the postnatal period	-	-	-	-	-	✵	-	-	-	-	-	-	✵	-
Advise pregnant women who have a sleep problem about sleep hygiene (including having a healthy bedtime routine, avoiding caffeine and reducing activity before sleep).	-	✵	✵	✵	-	✵	-	-	✵	✵	✵	-	✵	-
Offer women who have post-traumatic stress disorder, which has resulted from a traumatic birth, miscarriage, stillbirth or neonatal death, a high-intensity psychological intervention (trauma-focused CBT or eye movement desensitisation and reprocessing [EMDR])	-	✵	✵	-	-	✵	-	-	✵	✵	-	-	✵	-
Family therapy or couples focused work can be used to help families address any difficulties in the relationship or communication patterns.	-	✵	✵	-	-	-	✵	-	✵	✵	-	-	-	✵

	Antenatal							Postnatal						
	1	2	3	4	5	6	7	1	2	3	4	5	6	7
Equitable care considerations														
Discuss all treatment options that are appropriate and the availability of trained and local health-care providers. This will ensure that the individual is aware of the full spectrum of mental health services and supports, interventions, and potential outcomes. Support the individual's right to choice, the timing of care, and the selection of tailored approaches (where available)	-	✵	✵	✵	-	✵	-	-	✵	✵	✵	-	✵	-
Psychotherapies should be conducted by providers trained in the specific treatment(s). For example, CBT, IPT and PDT are effective in the treatment of PMHDs and may be used on their own or in combination.	✵	✵	✵	✵	-	✵	-	✵	✵	✵	✵	-	✵	-
Psychological and psychosocial interventions should be based on the relevant treatment manual(s), which should guide the structure and duration of the intervention.	-	-	-	-	-	✵	-	-	-	-	-	-	✵	-
Provide culturally relevant information on mental health problems in pregnancy and the postnatal period to the woman and, if she agrees, her partner, family or carer. Ensure that the woman understands that mental health problems are not uncommon during these periods and instil hope about treatment.	-	✵	✵	✵	-	✵	✵	-	✵	✵	✵	-	✵	✵
Individual and systemic considerations														
If a woman agrees, provide information to and involve her significant other(s) in discussions about her emotional wellbeing and care throughout the perinatal period.	✵	✵	✵	✵	-	✵	✵	✵	✵	✵	✵	-	✵	✵
Provide information and advice to enable the family member to support the mother	-	✵	✵	✵	-	✵	✵	-	✵	✵	✵	-	✵	✵
Include psychological preparation for parenthood as a routine part of antenatal care	-	-	✵	-	-	-	✵	-	-	-	-	-	-	-
**Mother-baby dyad**														
Therapeutic approaches in perinatal context														
Where there is evidence of impairment in the mother-infant relationship, additional interventions, specifically directed at that relationship, should be offered.	✵	✵	✵	-	-	✵	-	✵	✵	✵	-	-	✵	-

	Antenatal							Postnatal						
	1	2	3	4	5	6	7	1	2	3	4	5	6	7
Women with postnatal depression experiencing mother-infant difficulties – observation of mother-infant interactions, feedback, modelling and cognitive restructuring is recommended.	-	✸	-	-	-	-	-	-	✸	-	-	-	-	-
**Partners**														
Therapeutic approaches in perinatal context														
Consider how the partner's relationship with the baby can be supported alongside the mother’s care.	-	-	-	-	-	-	✸	-	-	-	-	-	-	✸
