# Supplementary material for: A systematic review of clinical psychological guidance for perinatal mental health
Source: BMC Psychiatry. 2023 Oct 30;23:790. doi: 10.1186/s12888-023-05173-1 (PMC10614401; doi:10.1186/s12888-023-05173-1)
Supplement: Supplementary file 4 — Additional file 4: Supplementary Table 4. Raw scores for the AGREE-II tool across each of the seven guidelines. [file 12888_2023_5173_MOESM4_ESM.pdf]

Supplementary table 4: Raw scores for the AGREE-II tool across each of the seven guidelines

		**DOMAINS**																							**Total score (max score = 161)**
		**Scope and purpose**			**Stakeholder involvement**			**Rigour of development**								**Clarity of presentation**			**Applicability**				**Editorial independence**		
		**ITEM**																							
	**Guideline organisation / society / publication year**	**1.** The overall objective(s) of the guideline is (are) specifically described.	**2.** The health question(s) covered by the guideline is (are) specifically described.	**3.** The population (patients, public, etc.) to whom the guideline is meant to apply is specifically described.	**4.** The guideline development group includes individuals from all relevant profession-al groups.	**5.** The views and preferences of the target population (patients, public, etc.) have been sought.	**6.** The target users of the guideline are clearly defined.	**7.** Systematic methods were used to search for evidence.	**8.** The criteria for selecting the evidence are clearly described	**9.** The strengths and limitations of the body of evidence are clearly described.	**10.** The methods for formulating the recommendations are clearly described.	**11.** The health benefits, side effects, and risks have been considered in formulating the recommendations.	**12.** There is an explicit link between the recommend-ations and the supporting evidence.	**13.** The guideline has been externally reviewed by experts prior to its publication	**14.** A procedure for updating the guideline is provided.	**15.** The recommend-ations are specific and unambiguous	**16.** The different options for manage-ment of the condition or health issue are clearly presented	**17.** Key recommend-ations are easily identifiable.	**18.** The guideline describes facilitators and barriers to its application.	**19.** The guideline provides advice and/or tools on how the recommendations can be put into practice.	**20.** The potential resource implications of applying the recommendations have been considered.	**21.** The guideline presents monitor-ing and/or auditing criteria.	**22.** The views of the funding body have not influenced the content of the guideline.	**23.** Competing interests of guideline develop-ment group members have been recorded and addressed.	
**1**	SIGN (2012)	7	7	7	5	5	7	4	5	5	4	5	6	5	5	5	6	7	1	4	5	5	1	6	**117**
**2**	Reproductive Mental Health Program & Perinatal Services BC (2014)	6	5	7	6	1	6	2	1	2	2	4	5	2	1	6	7	7	4	4	1	6	1	1	**87**
**3**	COPE (2017)	7	7	7	7	7	7	6	7	7	7	7	7	7	7	7	7	7	5	7	7	6	7	7	**157**
**4**	RNAO (2018)	7	7	7	7	7	6	7	6	5	5	7	7	7	7	7	7	7	5	5	5	5	7	6	**146**
**5**	Public Health Agency of Canada (2020)	6	7	7	6	1	5	7	7	7	2	7	5	3	1	7	7	4	7	7	1	4	1	1	**110**
**6**	NICE (2020)	7	7	7	7	7	7	6	7	7	6	7	5	6	1	5	7	6	4	5	5	5	1	7	**132**
**7**	NHS England (2021)	7	6	6	6	5	7	5	5	3	3	6	3	5	1	6	5	6	5	6	4	6	4	1	**111**
